# Facile Synthesis of MoP-RuP2 with Abundant Interfaces to Boost Hydrogen Evolution Reactions in Alkaline Media

**DOI:** 10.3390/nano11092347

**Published:** 2021-09-09

**Authors:** Zhi Chen, Ying Zhao, Yuxiao Gao, Zexing Wu, Lei Wang

**Affiliations:** State Key Laboratory Base of Eco-Chemical Engineering, College of Chemistry and Molecular Engineering, Qingdao University of Science & Technology, 53 Zhengzhou Road, Qingdao 266042, China; 2020050008@mails.qust.edu.cn (Z.C.); zhaoying99up@163.com (Y.Z.); 2020050019@mails.qust.edu.cn (Y.G.)

**Keywords:** electrocatalyst, hydrogen evolution reaction, interfaces

## Abstract

Exploiting efficient electrocatalysts for hydrogen evolution reactions (HERs) is important for boosting the large-scale applications of hydrogen energy. Herein, MoP-RuP_2_ encapsulated in N,P-codoped carbon (MoP-RuP_2_@NPC) with abundant interfaces were prepared via a facile avenue with the low-toxic melamine phosphate as the phosphorous resource. Moreover, the obtained electrocatalyst possessed a porous nanostructure, had abundant exposed active sites and improved the mass transport during the electrocatalytic process. Due to the above merits, the prepared MoP-RuP_2_@NPC delivered a greater electrocatalytic performance for HERs (50 mV@10 mA cm^−2^) relative to RuP_2_@NPC (120 mV) and MoP@NPC (195 mV) in 1 M KOH. Moreover, an ultralow potential of 1.6 V was required to deliver a current density of 10 mA cm^−2^ in the two-electrode configuration for overall water splitting. For practical applications, intermittent solar energy, wind energy and thermal energy were utilized to drive the electrolyzer to generate hydrogen gas. This work provides a novel and facile strategy for designing highly efficient and stable nanomaterials toward hydrogen production.

## 1. Introduction

Hydrogen is attracting significant attention around the world due to its high energy density, eco-friendly and sustainable merits, which favor alleviating the ever-increasing environmental pollution and energy crisis [1,2,3,4,5,6,7]. Among the developed technologies, electrochemical water splitting is regarded as an efficient and sustainable approach for hydrogen production [8,9,10,11,12,13,14]. As a half-reaction of water-splitting, the cathodic HER efficiency acts as a key role in reducing the energy consumption during hydrogen generation. Thus, it is of importance to develop electrocatalysts with advantageous catalytic performance for HERs [15,16,17,18,19,20,21]. At present, Pt-based nanomaterials are still the state-of-the-art electrocatalysts for HERs due to having the lowest overpotential and the smallest Tafel slope [22,23,24]. Unsatisfactorily, the scarcity and exorbitant price severely impede their widespread practical applications. Therefore, it is still worth developing alternatives to reduce the costs and accelerate the large-scale application of hydrogen energy in the actual environment [25,26].

Until now, a vast range of non-precious metal-based catalysts (e.g., metal sulfides [27,28,29], metal phosphides [30,31,32], metal carbides [33,34] and nonmetal-derived electrocatalysts [35,36]) have been investigated as substitutes for Pt for HERs [37,38]. However, compared with Pt-based electrocatalysts, the electrocatalytic activity of alternatives still lags [39]. More recently, tremendous efforts were made to explore other cost-effective precious metals (e.g., Pd, Ir and Ru) for promoting the HER catalytic activities due to their similar electron structures. Among them, ruthenium (Ru) is economically advantageous since the price is only one-fifteenth that of Pt. Moreover, the favorable Ru-H bond strength is also beneficial for boosting the reaction kinetics of HERs [40]. However, the electrocatalytic performance of Ru-derived nanomaterials still needs to improve further. As reported, the created interfaces in the developed electrocatalysts can regulate the adsorption/desorption of intermediates, tune the transportation of electrons and expose more active sites during electrocatalytic processes [41,42,43]. For instance, Fu and co-authors [44] constructed 2D MoP/MoS_2_ heterostructure nanosheets using two steps, namely, hydrothermal and phosphorization processes. The heterostructure catalyst helps to expose more active sites, thereby effectively activating H_2_O and achieving good electron transfer. Because of the above advantage, the obtained MoP/MoS_2_ presented an excellent electrocatalytic performance for HERs in neutral, alkaline and acid media. Structural and theoretical analysis results demonstrated that the surface of an MoP/MoS_2_ heterostructure accelerates H* and H_2_O adsorption due to the stronger interaction between MoS_2_ and MoP. Therefore, developing electrocatalysts with abundant interfaces is a promising avenue for boosting the electrocatalytic performance and lowering the content of noble metals.

**Scheme 1:**
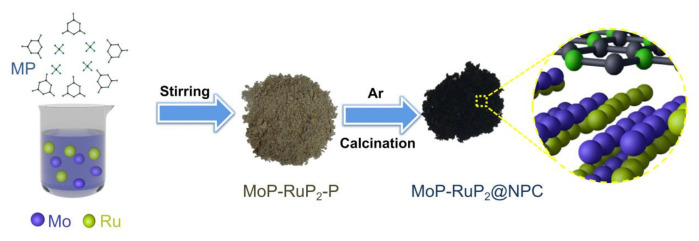
Schematic illustration of the synthesis of MoP-RuP_2_@NPC.

Herein, we describe the fabrication of MoP-RuP_2_@NPC with abundant interfaces via a facile one-step method, with the low-toxic melamine phosphate (MP) as the N and P resource (Scheme 1). The obtained electrocatalyst had rich interfaces and abundant pores, which allowed for the mass transport and acceleration of the reaction kinetics for HERs. Furthermore, the N,P-codoped carbon shell enhanced the electrochemical conductivity and protected against corrosion. Due to the above merits, the obtained MoP-RuP_2_@NPC presented remarkable catalytic activity and stability for HERs. Interestingly, the two-electrode setup was easily powered by sustainable energies, including solar, wind and biomass energies.

## 2. Materials and Methods

### 2.1. Synthesis of MoP@NPC

Melamine phosphate (MP, 224 mg) and (NH_4_)_6_Mo_7_O_24_·4H_2_O (58 mg) were uniformly dispersed in 20 mL of deionized water. The above solution was heated at 75 °C and stirred continuously until a viscous liquid was obtained. Then, the product was dried in a vacuum system for 12 h. Afterward, the as-prepared powder was annealed at different temperatures (700 °C, 800 °C and 900 °C) for 2 h under an Ar atmosphere with a flow rate of 2 °C min^−1^. RuP_2_@NCP was synthesized using the identical method with the addition of RuCl_3_ (11.5 mg). MoP-RuP_2_@NCP was synthesized in co-existence with RuCl_3_ (11.5 mg) and (NH_4_)_6_Mo_7_O_24_·4H_2_O (58 mg).

### 2.2. Physical Characterization

X-ray diffraction (XRD, X’Pert PRO) and X-ray photoelectron spectroscopy (XPS, AXIS SUPRA) were performed to identify the crystal structure and chemical composition. Scanning electron microscopy (SEM) and transmission electron microscopy (TEM) were conducted on a Regulus 8100 and a JEM-F200, respectively, to study the nanostructures of the prepared electrocatalysts.

### 2.3. Electrochemical Measurements

The electrocatalytic measurements were investigated using a three-electrode configuration with carbon rods, a reversible hydrogen electrode (RHE) and a glassy carbon electrode (5 mm in diameter), which were utilized as the counter electrodes, reference electrodes and working electrodes, respectively. A total of 5 mg of catalyst was dispersed in isopropanol solution (1 mL) with 20 µL Nafion solution (Shanghai, China 5 wt%) and then sonicated for 50 min to form a homogeneous ink. Then, 20 µL of the ink was dripped onto the glassy carbon electrode (containing 0.10 mg of catalyst). The electrocatalytic performance for HER was measured in a 1.0 M KOH medium. Linear sweep voltammograms (LSVs) were collected with a scanning rate of 5 mV s^−1^. Long-term durability measurements were conducted with a specific potential. All electrochemical studies were investigated at room temperature.

## 3. Results and Discussion

**Figure 1:**
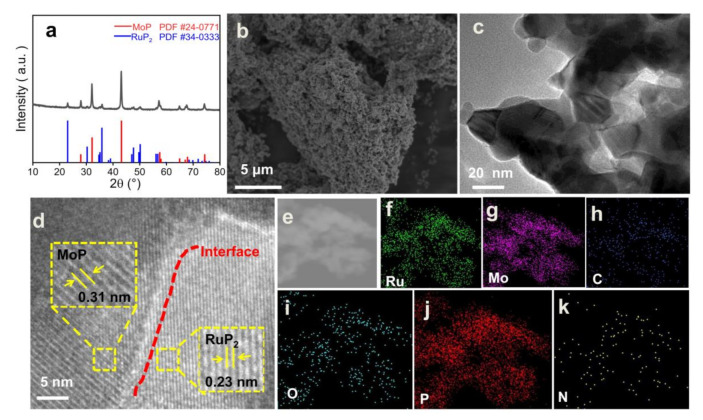
XRD (**a**), SEM (**b**) and TEM (**c**) images of MoP-RuP_2_@NPC; (**d**) HRTEM image; (**e**) TEM/EDS image; (**f**–**k**) elemental mapping images for Ru, Mo, C, O, P and N.

X-ray diffraction (XRD) measurements were used to characterize the structure of the prepared electrocatalyst. We identified that the designed MoP-RuP_2_@NPC was composed of MoP (PDF# 24-0771) and RuP_2_ (PDF# 34-0333) (Figure 1a). As reference samples, RuP_2_@NPC and MoP@NPC were composed of RuP_2_ and MoP, respectively (Figure S1). The morphology and structure of the obtained electrocatalyst were studied using scanning electron microscopy (SEM) and transmission electron microscopy (TEM). The panoramic SEM image (Figure 1b) shows that abundant interconnected pores existed in the designed nanomaterials, which could expose rich active sites and benefit the mass transport during the electrocatalytic process. The TEM images (Figure 1c) demonstrate that the developed MoP-RuP_2_@NPC was composed of numerous connected nanoparticles. In the high-resolution TEM image (Figure 1d), the layer distances of 0.31 nm and 0.23 nm were ascribed to the (001) and (111) lattice planes of MoP and RuP_2_, respectively, demonstrating the existence of MoP and RuP_2_ in the obtained electrocatalyst. Interestingly, obvious interfaces were observed in the prepared MoP-RuP_2_@NPC, which had a pivotal role in improving the electrocatalytic performance. The energy-dispersive X-ray spectrum (EDX) elemental mapping, shown in Figure 1e–k, revealed the homogenous distributions of Ru, Mo, C, O, P and N in the obtained MoP-RuP_2_@NPC.

**Figure 2:**
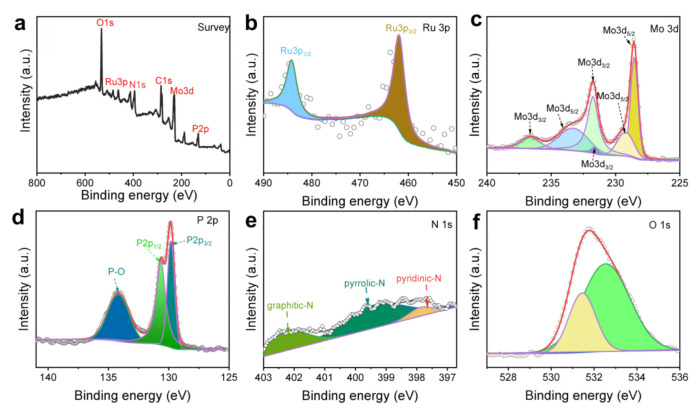
(**a**) The survey spectra for the MoP-RuP_2_@NPC. High-resolution XPS spectra of Ru 3p (**b**), Mo 3d (**c**), P 2p (**d**), N 1s (**e**) and O 1s (**f**) of the MoP-RuP_2_@NPC sample.

X-ray photoelectron spectroscopy (XPS) analyses were undertaken to further analyze the valence states and chemical composition. In line with the elemental mappings, the XPS survey spectra showed the existence of Ru, Mo, N, P, C and O elements in the prepared MoP-RuP_2_@NPC (Figure 2a). The high-resolution XPS spectrum of Ru showed peaks of Ru 2p3/2 and Ru 2p1/2 at 462 and 484.31 eV, respectively, are attributed to RuP_2_ [45,46], as shown in Figure 2b. For the high-resolution spectra of Mo 3d (Figure 2c), the binding energies at 228.5 and 231.80 eV could be assigned to Mo^δ+^ species (0 < δ < 4), which are normally connected with the molybdenum species in MoP [47]. The other weak peaks at 229.2, 231.7 eV and 233.5, 236.0 eV could be attributed to MoO_2_ and MoO_3_ species, respectively, owing to oxidation in the air condition [48]. The doublet peaks at 129.8 and 130.6 eV in the P 2p region could be assigned to metal–P bonds (Figure 2d) [49]. The other peak at 134.1 eV was attributed to P–O owing to oxidation after being exposed to air [50]. For the high-resolution XPS spectrum of N 1 s (Figure 2e), the 397.6, 399.5 and 402.14 eV peaks corresponded to pyridinic N, pyrrolic N and graphitic N, respectively [51]. The peaks centered at 533.1 eV and 531.6 eV were attributed to hydroxy oxygen, the physically adsorbed and chemisorbed water or other surface species in O 1s (Figure 2f) [52]. The atomic concentration of MoP-RuP2@NPC that was found using XPS (at%) is shown in Figure S2.

**Figure 3:**
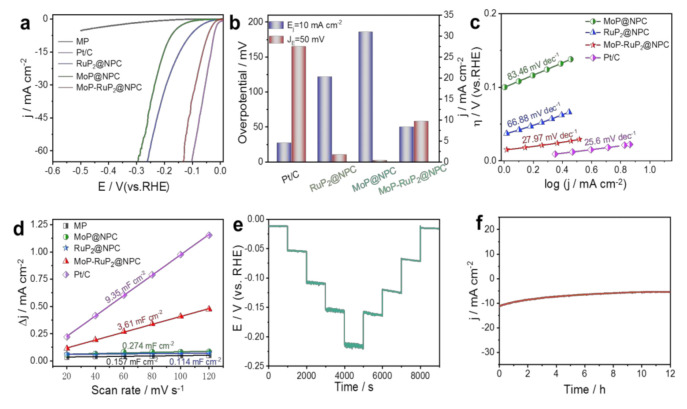
(**a**) LSVs of Pt/C, MoP-RuP_2_@NPC, MoP@NPC, RuP_2_@NPC and MP for the HERs with a scanning rate of 5 mV s^−1^ in 1 M KOH. (**b**) Summarized overpotentials (current density of 10 mA cm^−2^) and the current density (overpotential of 50 mV vs. RHE). (**c**) Tafel slopes and (**d**) Cdl of the obtained nanomaterials. (**e**) Multistep chronoamperometric curves of MoP-RuP_2_@NPC at various applied overpotentials in 1 M KOH. (**f**) Long-term stability study of the prepared electrocatalyst for 12 h.

The electrocatalytic HER performance of the prepared catalyst was estimated in a 1.0 M KOH electrolyte on a standard three-electrode configuration. The contents of the Ru and pyrolysis temperatures were first investigated on regulating the catalytic activities for the HERs (Figures S3 and S4). The MoP-RuP_2_@NPC with a molar ratio of 3:1 (Mo:Ru) at 800 °C presented the best catalytic performance for the HERs. As shown in Figure 3a, MoP-RuP_2_@NPC possessed the smallest overpotential of 50 mV to achieve 10 mA cm^−2^, relative to MoP@NPC (195 mV) and RuP_2_@NPC (120 mV), demonstrating that the coexistence of RuP_2_ and MoP was important for promoting the catalytic performances. The same tendency was also shown at a specific overpotential of 50 mV (Figure 3b). Moreover, the superior HER catalytic activity of MoP-RuP_2_@NPC was further investigated using the Tafel curve (Figure 3c).

The Tafel slope of MoP-RuP_2_@NPC was only 27.97 mV dec^−1^, much lower than MoP/NPC (83.46 mV dec^−1^) and RuP_2_@NPC (66.88 mV dec^−1^), demonstrating its rapid reaction kinetics for the HERs. Accordingly, the prepared MoP-RuP_2_@NPC possessed superior electrocatalytic performance for the HERs that was comparable with other previous nanomaterials (Table S1). The double-layer capacitance (Figure 3d) was related to the electrochemical surface areas (ECSA) of the designed electrocatalysts, which were investigated using capacitance measurements via cyclic voltammograms with different scan rates (Figure S5). The MoP-RuP_2_@NPC exhibited a Cdl value of 3.61 mF cm^−2^, which was higher than MoP@NPC (0.274 mF cm^−2^) and RuP_2_@NPC catalysts (0.114 mF cm^−2^), as well as MP (0.157 mF cm^−2^). The high Cdl value of MoP-RuP_2_@NPC implied the existence of abundant catalytic active sites, which led to its remarkable HER performance. Besides the catalytic activity, long-term durability is also an important factor for catalysts in practical applications. As illustrated in Figure 3e, the overpotential increased and remained high during the reversed process, indicating the excellent stability and mass transport properties. Moreover, MoP-RuP_2_@NPC presented excellent long-term durability at a current density of 10 mA cm^−2^ over 12 h (Figure 3f). Furthermore, MoP-RuP_2_@NPC showed a similar initial HER performance after 2000 cycles of a cyclic voltammetry test (Figure S6). These observations suggest that the MoP-RuP_2_@NPC possessed excellent stability for the HERs.

**Figure 4:**
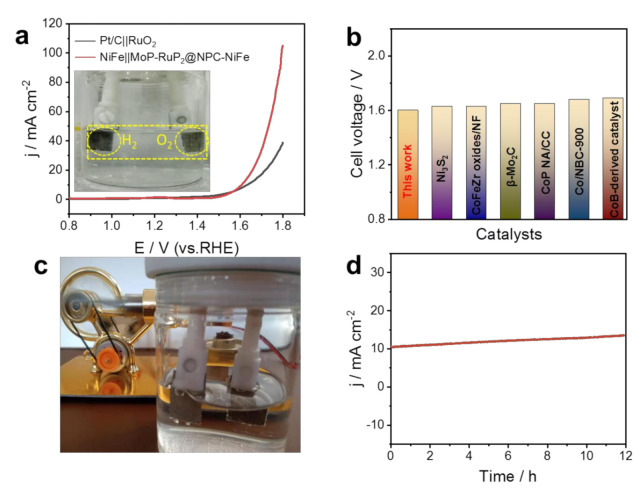
(**a**) Polarization curves of Pt/C||RuO_2_ and NiFe||MoP-RuP_2_@NPC-NiFe for overall water splitting in 1 M KOH. (**b**) Comparison of the overpotentials of the synthesized electrocatalysts with the reported values. (**c**) Electrocatalytic process powered using a Stirling engine. Chronoampermetry curve (**d**) of NiFe||MoP-RuP_2_@NPC-NiFe.

As MoP-RuP_2_@NPC displayed excellent electrocatalytic performance and stability for the HERs in alkaline solution, a water-splitting electrolyzer was constructed by using the designed electrocatalysts as the cathode and commercial NiFe foam as the anode to drive an overall water-splitting process. As displayed in Figure 4a, an ultralow potential of 1.6 V was required to deliver a current density of 10 mA cm^−2^ and the produced gases could be clearly observed in the picture (inset) and video (Movie S1). The electrolyzer composed of MoP-RuP_2_@NPC exhibited electrocatalytic performances that were comparable to preceding catalysts, such as Ni_3_S_2_ [53], CoFeZr oxides/NF [54], β-Mo_2_C [55], CoP NA/CC [56], Co/NBC-900 [57] and CoB-derived catalysts [58] (Figure 4b). Encouraged by the excellent catalytic performance, the electrolyzer setup with the developed MoP-RuP_2_@NPC as a cathode could be successfully driven by a Stirling engine, where a large number of bubbles were generated in the electrodes (Figure 4c and Movie S2). In order to further evaluate the practical application prospects, more tests were carried out and excellent performance was demonstrated. The designed electrolyzer could be powered using wind energy, a single AAA battery and solar energy (Figure S7 and Movies S3–S5). Furthermore, the long-term durability at a current density of 10 mA cm^−2^ was confirm by a negligible decay after 12 h of measurement, suggesting its excellent overall water-splitting stability (Figure 4d). Thus, the prepared electrocatalyst has potential in practical applications for hydrogen production. The excellent electrocatalytic performance could be attributed the following reasons: the abundant interfaces were important for boosting the catalytic performance; the interconnected pores allowed for favorable mass transport, exposing rich active sites and improving the release of generated bubbles; the heteroatom-doped carbon also contributed to enhancing the catalytic activity.

## 4. Conclusions

In summary, MoP-RuP_2_@NPC with abundant interfaces and rich pores was successfully prepared using a facile strategy with the low-toxic melamine phosphate as both the N and P source. The coupling effects and specific nanostructures endowed the designed nanomaterial with excellent electrocatalytic activity and long-term stability for the HERs. Moreover, MoP-RuP_2_@NPC exhibited a superior overall water-splitting performances with a low overpotential of 1.6 V to drive 10 mA cm^−2^. Moreover, sustainable energy sources, such as solar, thermal and wind, could be utilized to drive the electrolyzer, which could then can store the intermittent energies as hydrogen energy. This work opens a convenient and sustainable strategy to develop efficient electrocatalysts for hydrogen production.

## Data Availability

The data presented in this study are available on request from the corresponding author.

## Supplementary Materials

The following are available online at https://www.mdpi.com/article/10.3390/nano11092347/s1, Figure S1. XRD patterns of RuP2@NPC (a) and MoP@NPC (b), Figure S2. Atomic concentrations of MoP-RuP2@NPC in XPS (at%), Figure S3. LSVs of MoP-RuP2@NPC with various different content for HER in 1 M KOH, Figure S4. LSVs of MoP-RuP2@NPC with different temperatures for HER in 1 M KOH, Figure S5. CV curves of MoP-RuP2@NPC (a), MoP@NPC (b), RuP2@NPC (c), MP (d) and Pt/C at different scan rate in 1M KOH., Figure S6. Stability test for MoP-RuP2@NPC via CV scanning for 2000 cycles in 1 M KOH for HER, Figure S7. The diagram of the electrocatalytic overall water-splitting with the electric energy generated by wind (a), solar (b), battery (c), Table S1. Comparison of the electrocatalytic performance toward HER in 1 M KOH, Movie S1. The movie of the electrocatalytic overall water-splitting, Movie S2. Electrocatalytic process powered using a Stirling engine, Movie S3. The movie of the electrocatalytic overall water-splitting with the electric energy generated by battery, Movie S4. The movie of the electrocatalytic overall water-splitting with the electric energy generated by wind wind, Movie S5. The movie of the electrocatalytic overall water-splitting with the electric energy generated by wind solar.
