# Supplementary material for: Facile Synthesis of MoP-RuP2 with Abundant Interfaces to Boost Hydrogen Evolution Reactions in Alkaline Media
Source: Nanomaterials (Basel). 2021 Sep 9;11(9):2347. doi: 10.3390/nano11092347 (PMC8466548; doi:10.3390/nano11092347)
Supplement: Supplementary file 1 [file nanomaterials-11-02347-s001.zip › nanomaterials-1346955 - Supplementary Materials.pdf]

nanomaterials

MDPI

Supplementary Materials

Facile Synthesis of MoP-RuP2 with Abundant Interfaces to Boost Hydrogen Evolution Reactions in Alkaline Media

Zhi Chen, Ying Zhao, Yuxiao Gao, Zexing Wu * and Lei Wang *

State Key Laboratory Base of Eco-Chemical Engineering, College of Chemistry and Molecular Engineering, Qingdao University of Science & Technology, 53 Zhengzhou Road, Qingdao 266042, China; 2020050008@mails.qust.edu.cn (Z.C.); zhaoying99up@163.com (Y.Z.); 2020050019@mails.qust.edu.cn (Y.G.)

* Correspondence: splswzx@qust.edu.cn (Z.W.); inorchemwl@126.com (L.W.)

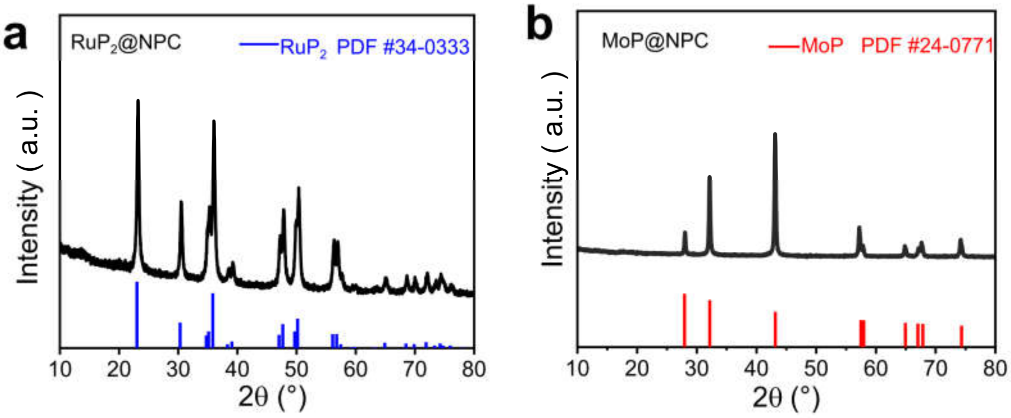

Figure S1. XRD patterns of RuP_2@NPC (**a**) and MoP@NPC (**b**).

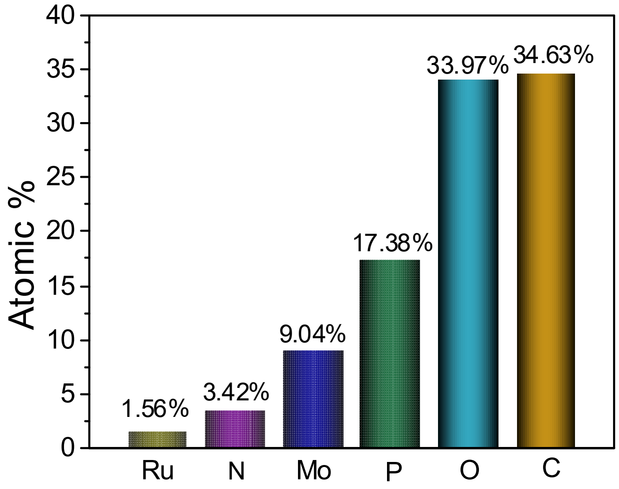

Figure S2. Atomic concentrations of MoP-RuP_2@NPC in XPS (at%).

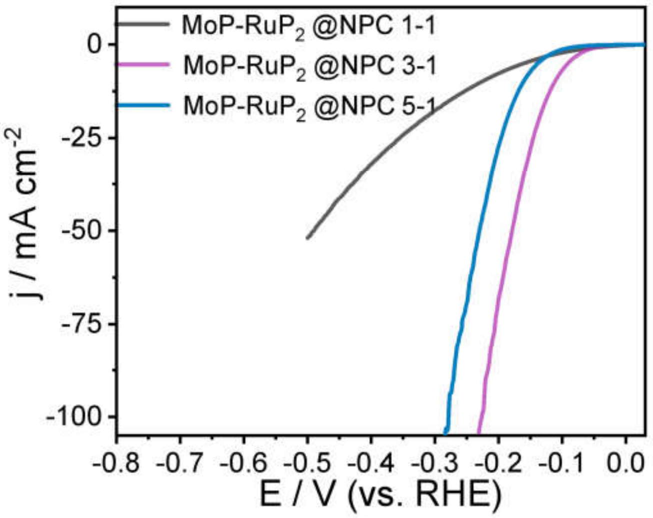

Figure S3. LSVs of MoP-RuP$_2$@NPC with various different content for HER in 1 M KOH.

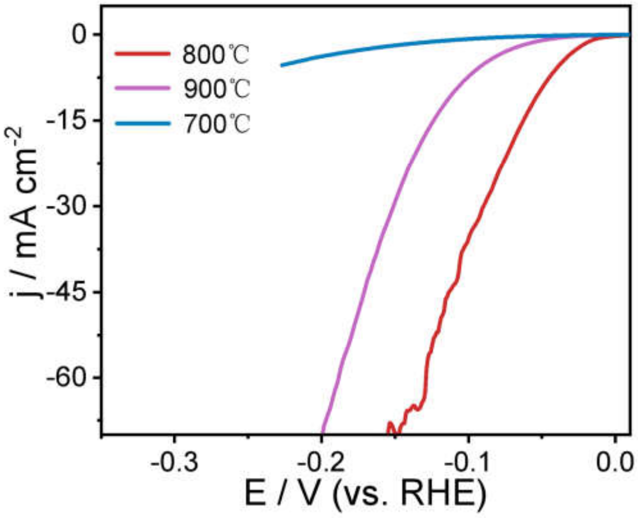

Figure S4. LSVs of MoP-RuP$_2$@NPC with different temperatures for HER in 1 M KOH.

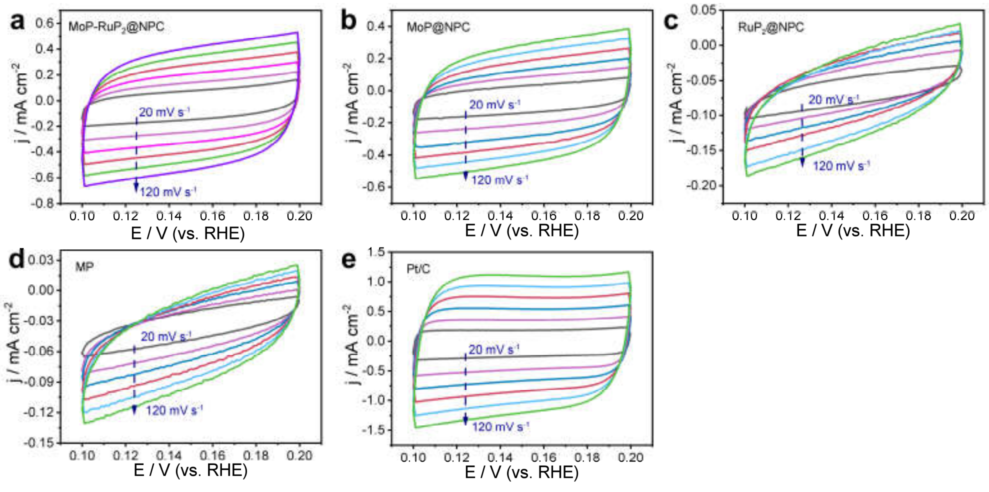

Figure S5. CV curves of MoP-RuP_2@NPC (**a**), MoP@NPC (**b**), RuP_2@NPC (**c**), MP (**d**) and Pt/C (**e**) at different scan rate in 1M KOH.

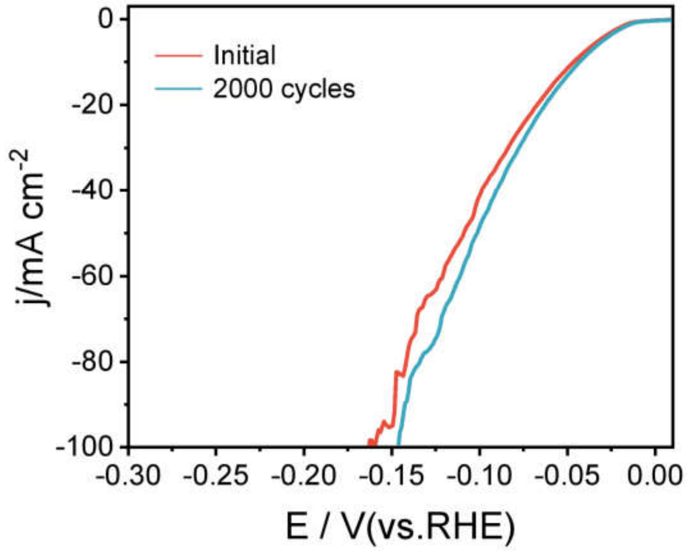

Figure S6. Stability test for MoP-RuP_2@NPC via CV scanning for 2000 cycles in 1 M KOH for HER.

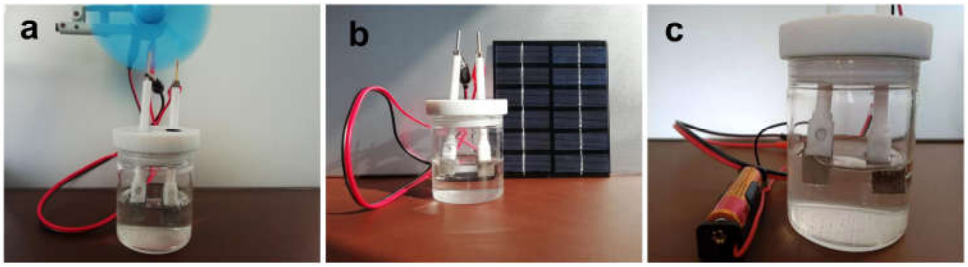

Figure S7. The diagram of the electrocatalytic overall water-splitting with the electric energy generated by wind (**a**), solar (**b**), battery (**c**).

Table S1. Comparison of the electrocatalytic performance toward HER in 1 M KOH.

Catalysts	Electrolyte	Overpotential@10 $mAcm^{-1}$ (mV)	Tafel Slope ($mV\ dec^{-1}$)	Reference
MoP-RuP_2/NPC	1M KOH	50	27.97	This work
Mo_2C/W_2C	1M KOH	132	76	1
Ni-S	1M KOH	58	81.6	2
Ni-Se-Cu	1M KOH	136	117.5	3
WS_2/CoS_2/CC	1M KOH	122	93	4
HEI	1M KOH	88.2	40.1	5
CoP nanoparticles	1M KOH	87	105	6
Ni-Fe-Sn	1M KOH	253	61.5	7
Ni-Co-P / NF	1M KOH	85	46	8
NF @ Ni_3S_2 @ NCNTs	1M KOH	93.89	54	9
MoS_2	1M KOH	117	38	10

References

1. Ling, Y.; Kazim, F. M. D. ; Zhang, Q.; Xiao, S.; Li, M.; Yang, Z. Construction of Mo_2C/W_2C heterogeneous electrocatalyst for efficient hydrogen evolution reaction. *Int. J. Hydrogen Energy*, 2021, **46**, 9699–9706.
2. Wu, Y.; Lian, J.; Wang, Y.; Sun, J.; He, Z.; Gu, Z. Potentiostatic electrodeposition of self-supported Ni S electrocatalyst supported on Ni foam for efficient hydrogen evolution. *Mater. Des.*, 2021, **198**.

3. Gao, Y.; Wu, Y.; He, H.; Tan, W. Potentiostatic electrodeposition of Ni-Se-Cu on nickel foam as an electrocatalyst for hydrogen evolution reaction. *J. Colloid Interface Sci.*, 2020, **578**, 555–564.
4. Wu, J.; Chen, T.; Zhu, C.; Du, J.; Huang, L.; Yan, J.; Cai, D.; Guan, C.; Pan, C. Rational Construction of a WS_2/CoS_2 Heterostructure Electrocatalyst for Efficient Hydrogen Evolution at All pH Values. *ACS Sustainable Chem. Eng.*, 2020, **8**, 4474–4480.
5. Jia, Z.; Yang, T.; Sun, L.; Zhao, Y.; Li, W.; Luan, J.; Lyu, F.; Zhang, L.; Kruzic, J. J.; Kai, J.; Huang, J.; Lu, J.; Liu, C. A Novel Multinary Intermetallic as an Active Electrocatalyst for Hydrogen Evolution. *Adv. Mater.*, 2020, **32**, e2000385.
6. Luo, S.; Hei, P.; Wang, R.; Yin, J.; Hong, W.; Liu, S.; Bai, Z.; Jiao, T. Facile synthesis of cobalt phosphide nanoparticles as highly active electrocatalysts for hydrogen evolution reaction. *Colloids Surf., A*, 2020, **600**.
7. Wu, Y.; Gao, Y.; He, H.; Zhang, P. Electrodeposition of self-supported Ni–Fe–Sn film on Ni foam: An efficient electrocatalyst for oxygen evolution reaction. *Electrochim. Acta*, 2019, **301**, 39–46.
8. Zhou, W.; Zhao, J.; Guan, J.; Wu, M.; Li, G. Ni_3S_2 in Situ Grown on Ni Foam Coupled with Nitrogen-Doped Carbon Nanotubes as an Efficient Electrocatalyst for the Hydrogen Evolution Reaction in Alkaline Solution. *ACS Omega*, 2019, **4**, 20244–20251.
9. Zhu, P.; Chen, Y.; Zhou, Y.; Yang, Z.; Wu, D.; Xiong, X.; Ouyang, F. A metallic MoS_2 nanosheet array on graphene-protected Ni foam as a highly efficient electrocatalytic hydrogen evolution cathode. *J. Mater. Chem. A*, 2018, **6**, 16458–16464.
10. Liu, Z.; Wang, J.; Zhan, C.; Yu, J.; Cao, Y.; Tu, J.; Shi, C. Phosphide-oxide honeycomb-like heterostructure $CoP@CoMoO_4/CC$ for enhanced hydrogen evolution reaction in alkaline solution. *J. Mater. Sci. Technol.*, 2020, **46**, 177–184.
